# Supplementary figures and images for: The Alzheimer’s gene SORL1 is a regulator of endosomal traffic and recycling in human neurons
Source: Cell Mol Life Sci. 2022 Feb 28;79(3):162. doi: 10.1007/s00018-022-04182-9 (PMC8885486; doi:10.1007/s00018-022-04182-9)

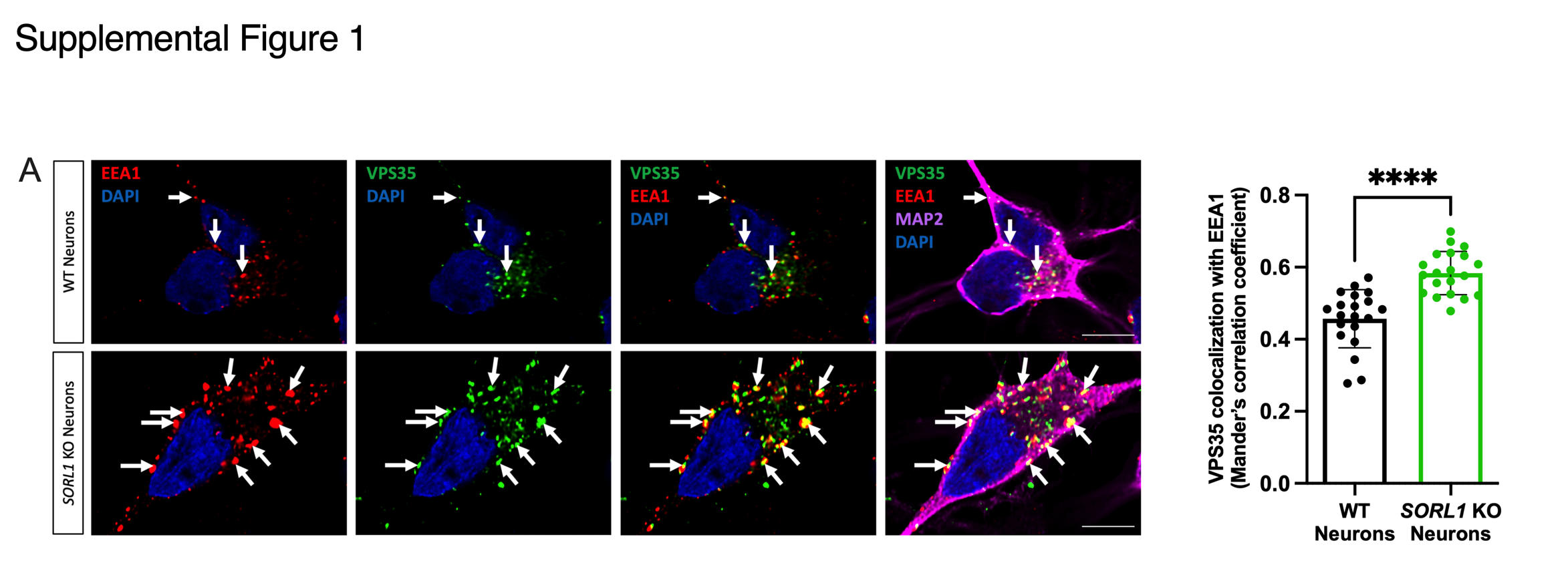

Supplement: Supplementary file 1 — Supplementary file1: Supplementary Fig. 1 Loss of SORL1 expression leads to increased VPS35 localization in early endosomes. (a) Representative immunofluorescent images of WT and SORL1KO neurons showing increased colocalization of VPS35 (green) with EEA1 (red). All neurons were immunolabeled with MAP2 (far-red) and counterstained with DAPI (blue). Scale bar: 10 μm. In all cases, quantification of colocalization was represented as Mander’s correlation co-efficient (MCC). 1 WT and 2 SORL1KO isogenic clones were used for these experiments and 10 images per clone per genotype were analyzed. Data represented as mean ± SD. Significance was determined using parametric two-tailed unpaired t test and was defined as a value of *p < 0.05, **p < 0.01, ***p < 0.001, and ****p < 0.0001. (TIFF 5815 KB) [file 18_2022_4182_MOESM1_ESM.tiff]

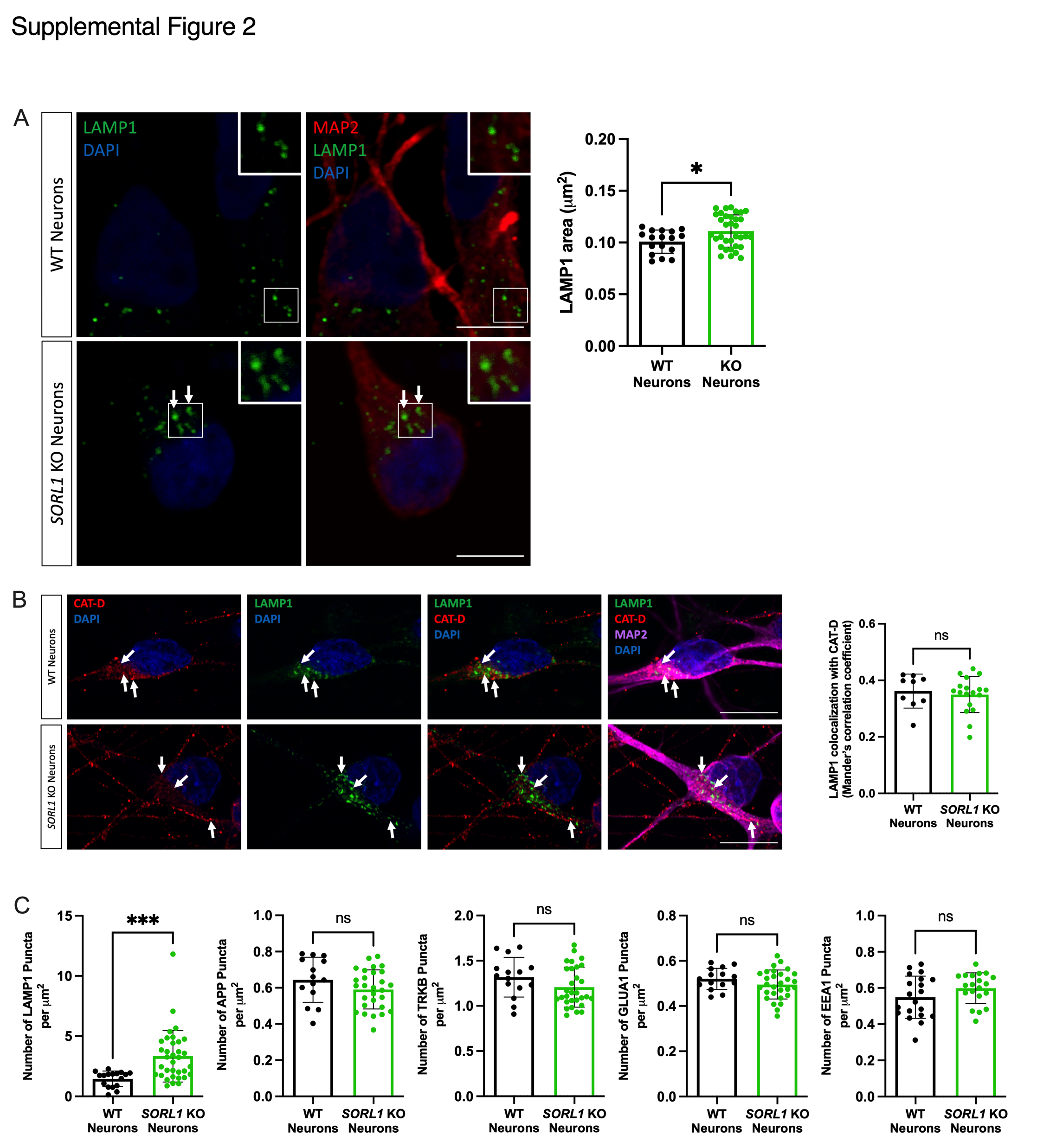

Supplement: Supplementary file 2 — Supplementary file2: Supplementary Fig. 2 a) SORL1KO neurons show larger lysosome size and increased lysosome number. Representative immunofluorescent images of WT and SORL1KO neurons labeled with LAMP1 (green) and MAP2 (red) showing increased LAMP1 positive vesicle size in SORL1KO neurons. Quantification of LAMP1 size and number was performed using Cell Profiler software. LAMP1 size is represented as area of LAMP1 positive vesicles, and LAMP1 number is represented as number of LAMP1 positive vesicles per square micron of cell area. Scale bar: 5 μm (b) SORL1KO neurons show no change in colocalization of lysosomes with the lysosomal enzyme Cathepsin D. Representative immunofluorescent images of WT and SORL1KO neurons labeled with antibodies for LAMP1 (green), Cathepsin D (red) and MAP2 (Far-red) showing no alteration in colocalization of Cathepsin-D with LAMP1 in SORL1KO neurons. Nuclei counterstained with DAPI (blue). Scale bar: 10 μm Quantification of colocalization of LAMP1 with Cathepsin-D represented as Mander’s correlation coefficient (MCC). 10–20 images were analyzed per genotype. Two isogenic clones of each genotype were used in all experiments. Data represented as mean ± SD. Normally distributed data was analyzed using parametric two-tailed unpaired t test. Significance was defined as a value of *p < 0.05, **p < 0.01, ***p < 0.001, and ****p < 0.0001. (TIFF 16410 KB) [file 18_2022_4182_MOESM2_ESM.tiff]

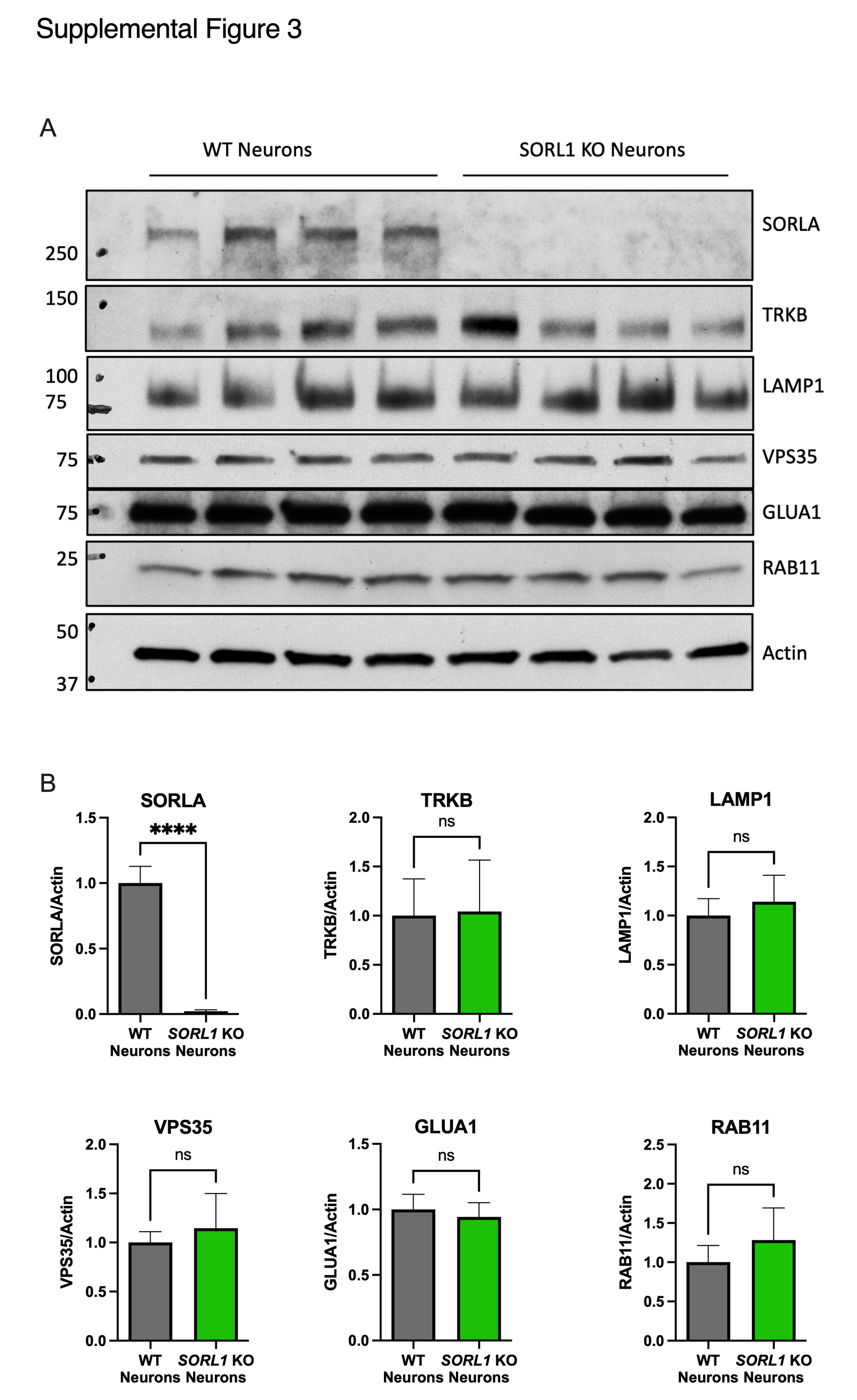

Supplement: Supplementary file 3 — Supplementary file3: Supplemental Fig. 3 Loss of SORL1 does not change protein expression of the compartments or cargo analyzed in this study as analyzed by Western blot. Representative blots in (a), quantification in (b). (TIFF 18476 KB) [file 18_2022_4182_MOESM3_ESM.tiff]

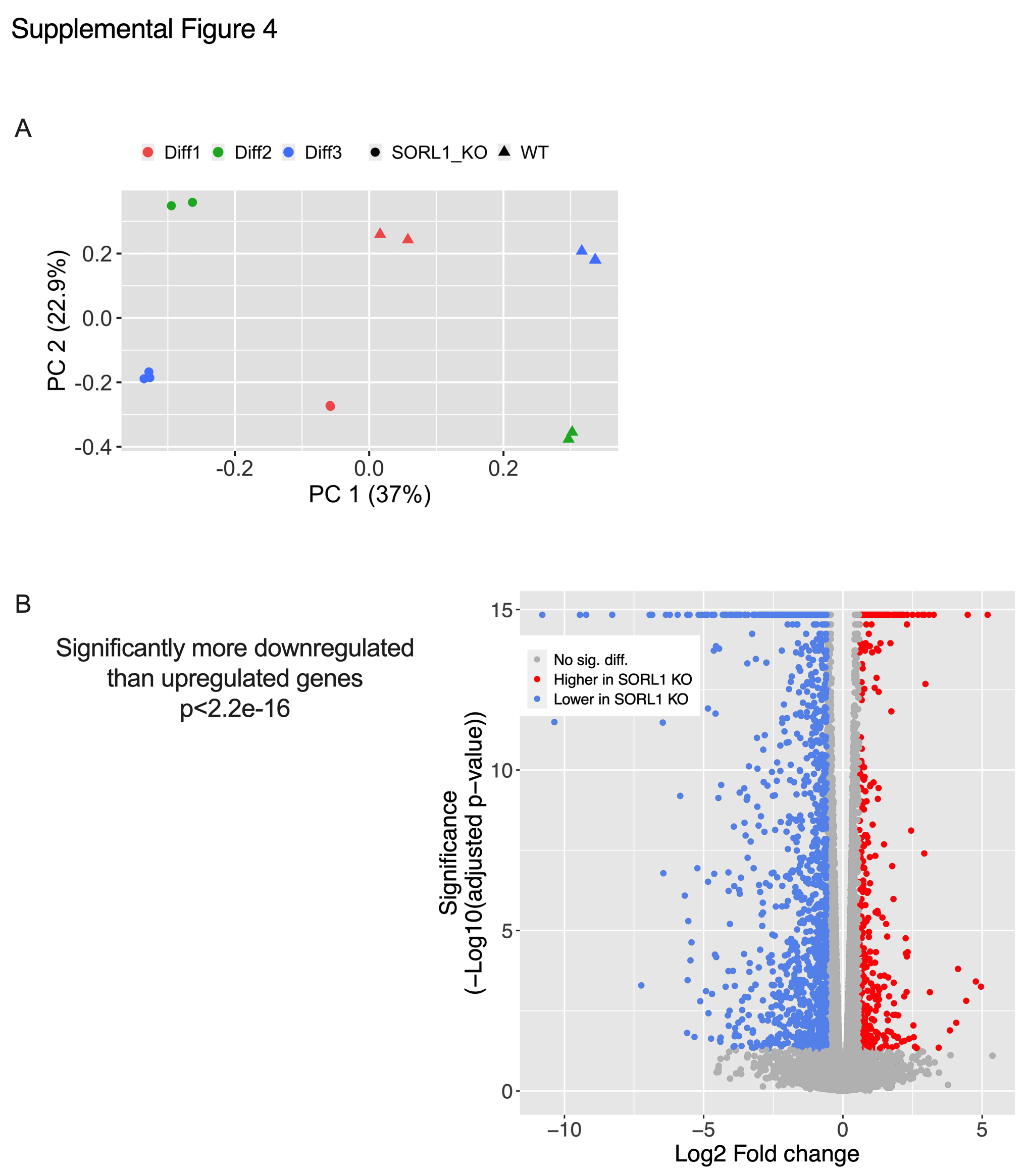

Supplement: Supplementary file 4 — Supplementary file4: Supplemental Fig. 4 a) Principal component analysis (PCA) plot of all RNAseq samples using all expressed genes. Samples are color coded by differentiation batch. Triangles represent WT samples, circles represent SORL1KO. Genotype accounts for the highest variance (37%, PC1, x-axis). b) Volcano plot. Log2 fold change between SORL1KO and WT is shown along the x-axis. Statistical significance is shown along the y-axis, and is measured by adjusted p-value. Genes upregulated in SORL1KO neurons are shown by red circles, genes downregulated in SORL1KO neurons are shown by blue circles. We observed 6643 DEGs, with 2819 upregulated and 3824 downregulated. There are significantly more down regulated genes than upregulated genes (p < 2.2e-16). Grey circles represent genes that are not significantly differentially expressed. (TIFF 13858 KB) [file 18_2022_4182_MOESM4_ESM.tiff]

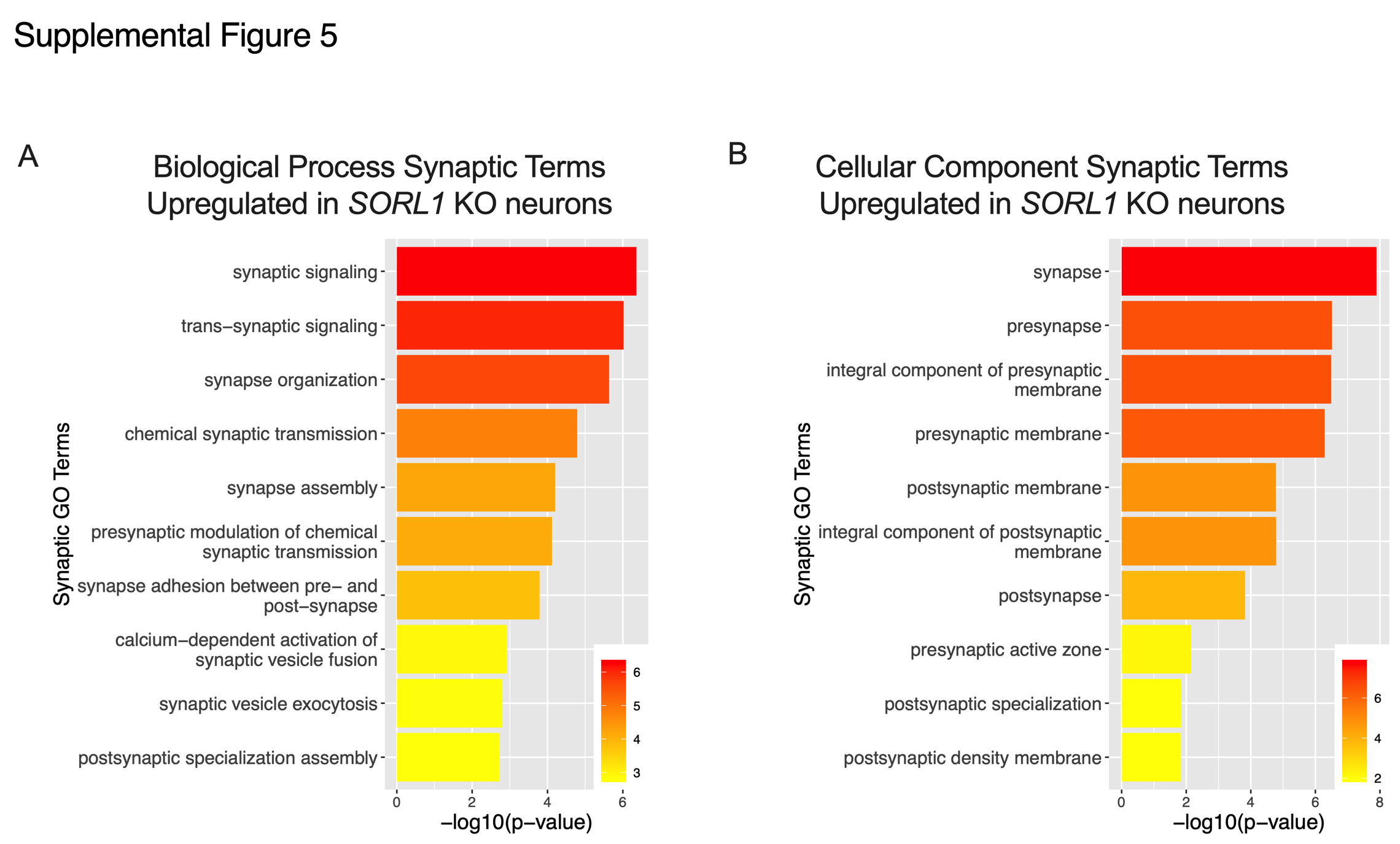

Supplement: Supplementary file 5 — Supplementary file5: Supplemental Fig. 5 Loss of SORL1 expression alters synaptic pathways. Analysis of bulk RNA-sequencing data indicates alterations in synaptic pathway functioning. We conducted gene ontology analysis of DEGs in WT and SORL1KO neurons using the SynGO synaptic annotation database. Shown here are the top upregulated and biological process and cellular component terms in SORL1KO neurons. GO annotation terms are listed on the y-axis, adjusted p-value is shown on the x-axis. No downregulated pathways in the SynGO database were shown to be enriched in SORL1KO neurons. (TIFF 12250 KB) [file 18_2022_4182_MOESM5_ESM.tiff]
