# Supplementary material for: The Alzheimer’s gene SORL1 is a regulator of endosomal traffic and recycling in human neurons
Source: Cell Mol Life Sci. 2022 Feb 28;79(3):162. doi: 10.1007/s00018-022-04182-9 (PMC8885486; doi:10.1007/s00018-022-04182-9)
Supplement: Supplementary file 6 — Supplementary file6: Supplemental Table 1 Summary of statistical analyses for data presented in this manuscript. In this table we present the statistical data that correspond to the experiments presented in the figures. This includes the group means, the difference between the means ± SEM, and the 95% confidence interval. (DOCX 22 KB) [file 18_2022_4182_MOESM6_ESM.docx]

| Figure Number | Experimental Measure | Group A | Group B | Group A Mean | Group B Mean | Difference between means  (B - A) ± SEM | 95% Confidence Interval |
| --- | --- | --- | --- | --- | --- | --- | --- |
| 1A | TRKB colocalization with EEA1 | WT Neurons | SORL1KO Neurons | 0.0799 | 0.1228 | 0.04290 ± 0.01042 | 0.02155 to 0.06425 |
| 1B | GLUA1 colocalization with EEA1 | WT Neurons | SORL1KO Neurons | 0.0496 | 0.1727 | 0.1231 ± 0.02257 | 0.07682 to 0.1693 |
| 2A | 6hr DQ Red BSA Intensity | WT Neurons | SORL1KO Neurons | 52.1 | 29.48 | -22.62 ± 2.684 | -28.09 to -17.15 |
| 2A | 24hr DQ Red BSA Intensity | WT Neurons | SORL1KO Neurons | 62.54 | 43.35 | -19.19 ± 3.608 | -26.58 to -11.80 |
| 2B | TRKB colocalization with RAB7 | WT Neurons | SORL1KO Neurons | 0.3102 | 0.2149 | -0.09530 ± 0.01234 | -0.1212 to -0.06937 |
| 2C | GLUA1 colocalization with RAB7 | WT Neurons | SORL1KO Neurons | 0.3828 | 0.286 | -0.09685 ± 0.02779 | -0.1538 to -0.03992 |
| 2D | APP colocalization with LAMP1 | WT Neurons | SORL1KO Neurons | 0.2983 | 0.1799 | -0.1184 ± 0.01951 | -0.1584 to -0.07844 |
| 2E | TRKB colocalization with LAMP1 | WT Neurons | SORL1KO Neurons | 0.071 | 0.04461 | -0.02639 ± 0.004876 | -0.03643 to -0.01635 |
| 2F | GLUA1 colocalization with LAMP1 | WT Neurons | SORL1KO Neurons | 0.1967 | 0.1588 | -0.03790 ± 0.02085 | -0.08060 to 0.004801 |
| 3A | 6hr DQ Red BSA Intensity | WT Neurons | SORL1OE Neurons | 30.31 | 27.84 | -2.473 ± 2.059 | -6.799 to 1.854 |
| 3A | 24hr DQ Red BSA Intensity | WT Neurons | SORL1OE Neurons | 32.61 | 39.73 | 7.123 ± 1.887 | 3.157 to 11.09 |
| 3B | APP colocalization with RAB7 | WT Neurons | SORL1OE Neurons | 0.7041 | 0.8008 | 0.09670 ± 0.02520 | 0.04375 to 0.1496 |
| 3C | TRKB colocalization with RAB7 | WT Neurons | SORL1OE Neurons | 0.44 | 0.5261 | 0.08610 ± 0.02417 | 0.03532 to 0.1369 |
| 3D | GLUA1 colocalization with RAB7 | WT Neurons | SORL1OE Neurons | 0.4287 | 0.5512 | 0.1225 ± 0.02485 | 0.07029 to 0.1747 |
| 3E | APP colocalization with LAMP1 | WT Neurons | SORL1OE Neurons | 0.1397 | 0.2949 | 0.1552 ± 0.02066 | 0.1118 to 0.1986 |
| 3F | TRKB colocalization with LAMP1 | WT Neurons | SORL1OE Neurons | 0.1539 | 0.2674 | 0.1135 ± 0.01501 | 0.08196 to 0.1450 |
| 3G | GLUA1 colocalization with LAMP1 | WT Neurons | SORL1OE Neurons | 0.1353 | 0.3206 | 0.1853 ± 0.02279 | 0.1374 to 0.2332 |
| 4A - T0 | Transferrin Intensity T0 | WT Neurons - T0 | SORL1KO Neurons - T0 | 100 | 100 | -4.167E-08 | -4.634 to 4.634 |
| 4A - T10 | Transferrin Intensity T10 | WT Neurons - T10 | SORL1KO Neurons - T10 | 64.14 | 82.82 | -18.68 | -24.35 to -13.00 |
| 4A - T20 | Transferrin Intensity T20 | WT Neurons - T20 | SORL1KO Neurons - T20 | 58.44 | 67.48 | -9.038 | -13.67 to -4.404 |
| 4A - T40 | Transferrin Intensity T40 | WT Neurons - T40 | SORL1KO Neurons - T40 | 56.41 | 68.96 | -12.55 | -17.19 to -7.920 |
| 4B | Rab11 Area | WT Neurons | SORL1KO Neurons | 0.06625 | 0.073 | 0.006747 ± 0.002882 | 0.0009338 to 0.01256 |
| 4C | APP colocalization with RAB11 | WT Neurons | SORL1KO Neurons | 0.2689 | 0.3147 | 0.04580 ± 0.01442 | 0.01626 to 0.07534 |
| 4D | TRKB colocalization with RAB11 | WT Neurons | SORL1KO Neurons | 0.1071 | 0.2277 | 0.1206 ± 0.02924 | 0.06071 to 0.1805 |
| 4E | GLUA1 colocalization with RAB11 | WT Neurons | SORL1KO Neurons | 0.2605 | 0.3065 | 0.04595 ± 0.01599 | 0.01320 to 0.07870 |
| 5A | Surface:Total APP Intensity | WT Neurons | SORL1KO Neurons | 1.128 | 0.5124 | -0.6157 ± 0.05825 | -0.7331 to -0.4982 |
| 5B | Surface:Total GLUA1 Intensity | WT Neurons | SORL1KO Neurons | 1.022 | 0.5769 | -0.4451 ± 0.04588 | -0.5368 to -0.3534 |
| 5C | D27 Weighted Mean Firing | WT Neurons | SORL1KO Neurons | 3.768 | 7.696 | 3.928 ± 1.266 | 1.169 to 6.687 |
| 5C | D66 Weighted Mean Firing | WT Neurons | SORL1KO Neurons | 7.074 | 3.784 | -3.289 ± 1.101 | -5.688 to -0.8903 |
| 6A - T0 | Transferrin Intensity T0 | WT Neurons - T0 | SORL1OE Neurons - T0 | 100 | 100 | -0.0000002 | -11.47 to 11.47 |
| 6A - T10 | Transferrin Intensity T10 | WT Neurons - T10 | SORL1OE Neurons - T10 | 82.52 | 69.57 | 12.95 | 1.483 to 24.42 |
| 6A - T20 | Transferrin Intensity T20 | WT Neurons - T20 | SORL1OE Neurons - T20 | 74.47 | 59.35 | 15.12 | 3.653 to 26.59 |
| 6A - T40 | Transferrin Intensity T40 | WT Neurons - T40 | SORL1OE Neurons - T40 | 68.31 | 55.43 | 12.89 | 1.419 to 24.35 |
| 6B | Rab11 Area | WT Neurons | SORL1OE Neurons | 0.101 | 0.09005 | -0.01099 ± 0.003199 | -0.01742 to -0.004558 |
| 6C | APP colocalization with RAB11 | WT Neurons | SORL1OE Neurons | 0.3903 | 0.5878 | 0.1975 ± 0.02717 | 0.1404 to 0.2546 |
| 6D | TRKB colocalization with RAB11 | WT Neurons | SORL1OE Neurons | 0.1846 | 0.2969 | 0.1123 ± 0.02416 | 0.06155 to 0.1631 |
| 6E | GLUA1 colocalization with RAB11 | WT Neurons | SORL1OE Neurons | 0.2509 | 0.2965 | 0.04560 ± 0.01822 | 0.007315 to 0.08389 |
| 6F | Surface:Total APP Intensity | WT Neurons | SORL1OE Neurons | 0.9734 | 1.189 | 0.2155 ± 0.09083 | 0.02710 to 0.4038 |
| 6G | Surface:Total GLUA1 Intensity | WT Neurons | SORL1OE Neurons | 0.5727 | 0.9505 | 0.3778 ± 0.06831 | 0.2374 to 0.5182 |
| Supplementary 1A | VPS35 colocalization with EEA1 | WT Neurons | SORL1KO Neurons | 0.457 | 0.5838 | 0.1268 ± 0.02249 | 0.08129 to 0.1724 |
| Supplementary 2A | LAMP1 area | WT Neurons | SORL1KO Neurons | 0.1008 | 0.1112 | 0.01032 ± 0.004276 | 0.001730 to 0.01892 |
| Supplementary 2B | Cathepsin-D colocalization with LAMP1 | WT Neurons | SORL1KO Neurons | 0.3621 | 0.3499 | -0.01219 ± 0.02558 | -0.06488 to 0.04051 |
| Supplementary 2C | Number of LAMP1 puncta per µm^2^ | WT Neurons | SORL1KO Neurons | 1.452 | 3.339 | 1.887 ± 0.5339 | 0.8145 to 2.960 |
| Supplementary 2C | Number of APP puncta per µm^2^ | WT Neurons | SORL1KO Neurons | 0.6442 | 0.5906 | -0.05352 ± 0.03725 | -0.1288 to 0.02175 |
| Supplementary 2C | Number of TRKB puncta per µm^2^ | WT Neurons | SORL1KO Neurons | 1.318 | 1.208 | -0.1100 ± 0.06986 | -0.2509 to 0.03084 |
| Supplementary 2C | Number of GLUA1 puncta per µm^2^ | WT Neurons | SORL1KO Neurons | 0.5202 | 0.4951 | -0.02509 ± 0.01880 | -0.06303 to 0.01285 |
| Supplementary 2C | Number of EEA1 puncta per µm^2^ | WT Neurons | SORL1KO Neurons | 0.5488 | 0.5986 | 0.04975 ± 0.03228 | -0.01560 to 0.1151 |
| Supplementary 3B | SORLA/Actin | WT Neurons | SORL1KO Neurons | 1 | 0.02221 | -0.9778 ± 0.06426 | -1.135 to -0.8205 |
| Supplementary 3B | TRKB/Actin | WT Neurons | SORL1KO Neurons | 1 | 1.043 | 0.04315 ± 0.3219 | -0.7444 to 0.8307 |
| Supplementary 3B | LAMP1/Actin | WT Neurons | SORL1KO Neurons | 1 | 1.141 | 0.1413 ± 0.1599 | -0.2499 to 0.5324 |
| Supplementary 3B | VPS35/Actin | WT Neurons | SORL1KO Neurons | 1 | 1.146 | 0.1459 ± 0.1848 | -0.3064 to 0.5982 |
| Supplementary 3B | GLUA1/Actin | WT Neurons | SORL1KO Neurons | 1 | 0.9428 | -0.05717 ± 0.07946 | -0.2516 to 0.1373 |

Supplemental Table 1.
